# A Chlorhexidine Nanocarrier Strategy to Combat Oral Candidiasis Microcosm Biofilms

**DOI:** 10.3390/ph18111597

**Published:** 2025-10-22

**Authors:** Leandro Pimentel Cabral, Juliano Pelim Pessan, Caio Sampaio, Rosana Leal do Prado, Thayse Yumi Hosida, Celso Koogi Sonoda, Douglas Roberto Monteiro

**Affiliations:** 1 Department of Pediatric Dentistry and Public Health, School of Dentistry, Araçatuba, São Paulo State University (UNESP), Araçatuba 16015-050, São Paulo, Brazil; leandro.cabral@unesp.br (L.P.C.); juliano.pessan@unesp.br (J.P.P.); caio.sampaio@unesp.br (C.S.); thayse.hosida@unesp.br (T.Y.H.); 2 Department of Community and Preventive Dentistry, School of Dentistry, Federal University of Minas Gerais (UFMG), Belo Horizonte 31270-901, Minas Gerais, Brazil; rosanahb@yahoo.com.br; 3 Department of Diagnosis and Surgery, School of Dentistry, Araçatuba, São Paulo State University (UNESP), Araçatuba 16015-050, São Paulo, Brazil; celso.k.sonoda@unesp.br

**Keywords:** biofilms, chitosan, chlorhexidine, iron oxide nanoparticles, oral candidiasis

## Abstract

**Background/Objectives**: Nanotherapies are a strategy to combat *Candida* resistance. This study analyzed the impacts of iron oxide nanoparticles (IONPs) functionalized with a chitosan (CS) layer acting as carriers of chlorhexidine (CHX) on an oral candidiasis microcosm biofilm. **Methods**: Saliva samples from three healthy donors were used to form biofilms, to which *Candida* species were added to reproduce an oral candidiasis microcosm. Biofilms were cultivated for 72 h on glass coverslips using an active adhesion model. Biofilms without *Candida* served as a control model. The nanocarrier loaded with CHX at 78 (IONPs-CS-CHX78) or 156 µg/mL (IONPs-CS-CHX156) was co-incubated with the biofilms for 24 h. Controls included isolated IONPs, CS, and CHX, in addition to an untreated group (NC). Assays for biomass production, metabolism, microbial load, and lactic acid production were conducted to assess antibiofilm effects. Biofilm structure, viability, and thickness were also examined by confocal microscopy. Statistical analysis was performed using one-way ANOVA or Kruskal–Wallis, subsequently accompanied by the Student–Newman–Keuls post hoc test (*p* < 0.05). **Results**: CHX and IONPs-CS-CHX156 were the most effective agents against all tested biofilm models, significantly reducing metabolism, microbial load (bacterial and fungal), and viability. For the oral candidiasis biofilm, the nanocarrier did not affect biomass or biofilm thickness but led to a significant increase in lactic acid levels compared to NC. **Conclusions**: It is concluded that the nanocarrier of CHX exhibits a significant reducing effect on oral candidiasis microcosm biofilms at half the concentration required for non-carried CHX. This nanostructure can be explored in the development of antiseptic or disinfectant solutions for managing oral candidiasis.

## 1. Introduction

Oral candidiasis is an immunocompromised host fungal infection typically attributed to *Candida* spp. [1]. It is estimated that 30 to 60% of healthy adults have some *Candida* species in their oral microbiome, especially in the commensal state [2]. The transition to the pathogenic state, however, occurs when there is an imbalance in the interactions between host and pathogen. This may be caused by metabolic changes, indiscriminate or continuous use of antibiotics and steroids, weakened immune system, radioactive therapies, organ transplants, and xerostomia [3,4]. *Candida albicans* is the fungus most commonly linked to episodes of oral candidiasis, accounting for 95% of these [1]. In recent years, the use of medications has influenced the pathogenesis of this oral disease, leading to the growth of non-*albicans Candida* species, particularly *Candida glabrata*, with an increase in more than 17% in the number of cases of candidiasis caused by this fungal species [5].

Among the main virulence determinants of *Candida* species related to the oral candidiasis development are adhesion to different types of surfaces (oral tissues, rehabilitation materials, and orthodontic appliances) and the subsequent formation of microbial biofilms [6]. Fungal cells within biofilms exhibit higher pathogenicity than their counterparts in the planktonic state, mainly due to greater resistance to both traditional antifungals and phagocytosis by host immune cells [7]. Furthermore, when microorganisms compose polymicrobial biofilms, the community becomes more dynamic and difficult to eliminate compared to mono-species biofilms, particularly due to greater resistance to antimicrobials [8]. Resistance of *Candida* strains to conventional therapies poses a public health challenge. Fungal infections, including those associated with biofilm formation on medical devices and biologic tissues, can contribute to morbidity and mortality in immunocompromised individuals. The presence of resistant strains in fungal biofilms may also lead to persistent infections and treatment failure [9].

Regarding the management of oral candidiasis patients, topical antifungals are used to treat conditions of mild to moderate intensity [10]. In more serious situations or when topical therapy does not achieve the desired efficacy, the use of systemic antifungals is recommended, such as azoles (fluconazole and itraconazole) and polyenes (amphotericin B) [11]. However, the use of systemic antifungals is limited, mainly due to the lower susceptibility of some *Candida* species to azoles and the risk of interactions with other drugs, such as anticoagulants [12]. In this sense, a complementary alternative to antifungals in the treatment of oral candidiasis is chlorhexidine (CHX) [13], which is a cationic biguanide with antimicrobial activity against multiple pathogens [14]. The antifungal action of CHX on *C. albicans* cells is related to apoptosis due to the generation of reactive oxygen species (ROS), metabolic change, and disturbance of the homeostatic stability of metal ions [15,16]. Despite the high efficacy of CHX against several *Candida* species [17], some fungal strains may survive after exposure to CHX, generating a multitolerant subpopulation and the consequent reduction in the effectiveness of the product over time [18]. In addition, prolonged use of CHX may cause taste changes, dental stains, and changes in soft tissues, in addition to favouring the formation of supragingival calculus [19].

Given the scenario reported above, the development of alternative compounds to traditional antimicrobials is essential to provide a safer and more effective option for the management of oral candidiasis. Within this perspective, innovations in nanotechnology have allowed the improvement of multifunctional nanosystems capable of maximizing the molecular stability of drugs and their biological therapeutic efficacy [20,21]. Over the past few years, magnetic iron oxide nanoparticles (IONPs) have been widely evaluated in several innovative health-related applications [22]. One of these applications is their use as drug carriers to control microbial biofilms [23]. IONPs have low toxicity and can be easily conjugated with various drugs [23]. They also penetrate the matrix of microbial biofilms and can be directed to the infection site with an external magnetic field [23]. To perform the carrier function, the magnetite (Fe_3_O_4_) or maghemite (y-Fe_2_O_3_) core of IONPs is normally coated with a protective and functionalizing layer formed by various materials, including chitosan (CS), which is a natural glucosamine polymer resulting from the deacetylation of chitin [23,24]. CS has antimicrobial and mucoadhesive properties and is considered biocompatible [23]. A recent investigation revealed that binding CHX to magnetic nanoparticles intensifies the antimicrobial activity of the antiseptic on bacteria in the planktonic state and allows its controlled release, making this nanocarrier system an innovative approach for applications in the medical and dental areas [25]. Furthermore, aminosilane-coated IONPs loaded with CHX were more effective than unloaded CHX in combating microorganisms in the free state or organized as biofilms [26].

A CHX nanocarrier based on CS-coated IONPs was developed to enhance its efficacy against oral biofilms and to overcome *Candida* resistance [27,28]. The compound combines the beneficial characteristics of each material: IONPs enable drug delivery, CS enhances biological properties, and CHX acts as a potent broad-spectrum antimicrobial agent. When CS-coated and CHX-functionalized IONPs were tested on single or mixed biofilms formed by pathogens of dental interest, antibiofilm results equivalent to or more effective than those of unloaded CHX were demonstrated [27,28]. Despite the relevant results previously reported, the impacts of a nanocarrier of CHX inspired by CS-coated IONPs on polymicrobial biofilms originating from human saliva and developed by the active adhesion of microorganisms have not yet been revealed. Therefore, the current research intended to analyze the impacts of CS-coated IONPs operating as CHX carriers on salivary polymicrobial biofilm reproducing a microcosm of oral candidiasis and cultivated in an active adhesion model. The hypotheses tested in this study were that (i) the nanocarrier would significantly reduce the microcosm biofilms, and (ii) the nanocarrier load with CHX at half the concentration would demonstrate similar antibiofilm performance as free CHX.

## 2. Results

### 2.1. Salivary Microcosm Biofilm Without Candida spp.

**Figure 1:**
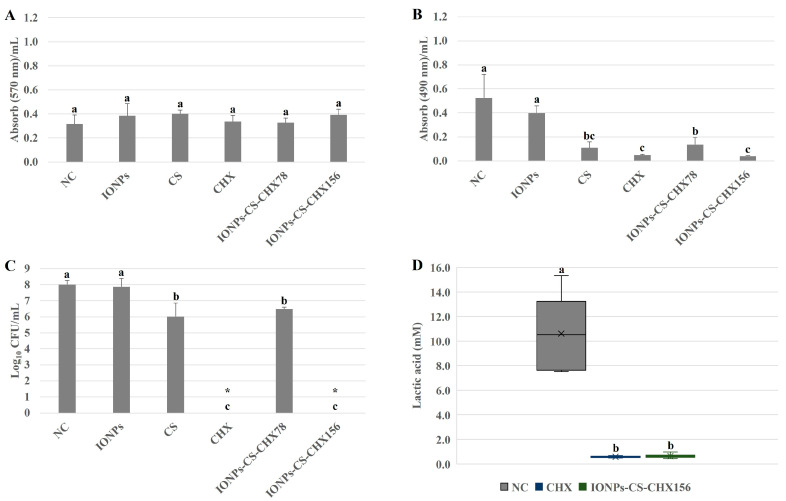
Semi-quantitative and quantitative assays of total biomass (**A**), metabolism (**B**), bacterial load (**C**), and lactic acid production (**D**) of salivary microcosm biofilms without *Candida* spp. in the various experimental groups. Seventy-two-hour biofilms remained in contact with the following agents for 24 h: pure McBain medium (negative control (NC)), iron oxide nanoparticles (IONPs) and chitosan (CS), both at 218.75 µg/mL, chlorhexidine at 312 μg/mL (CHX), and nanocarrier loaded with CHX at 78 (IONPs-CS-CHX78) or 156 μg/mL (IONPs-CS-CHX156). * Indicates complete eradication of cultivable cells. Groups differ by distinct lowercase letters (one-way ANOVA or Kruskal–Wallis test and Student-Newman-Keuls post hoc method; *p* < 0.05).

For the total biomass, none of the agents resulted in significant decreases in relation to the negative control (NC), and all evaluated treatments had similar effects (Figure 1A). Treatments of the salivary microcosm biofilm with CS, CHX, IONPs-CS-CHX78, and IONPs-CS-CHX156 resulted in significant reductions in metabolism of 78.84 (*p* < 0.001), 90.38 (*p* < 0.001), 73.07 (*p* < 0.001), and 92.30% (*p* < 0.001) in relation to NC, respectively (Figure 1B). No differences were observed among CS, CHX, and IONPs-CS-CHX156 concerning the impact on metabolic activity (Figure 1B).

Significant reductions in total bacterial load of 1.99- (*p* = 0.001) and 1.53-log_10_ (*p* = 0.004) were observed for CS and IONPs-CS-CHX78, respectively, compared to NC (Figure 1C). In turn, CHX and IONPs-CS-CHX156 provided the most expressive effects, reaching reductions of 8.01-log_10_ (*p* < 0.001) in colony counts compared to NC (Figure 1C). Equivalently, there was no significant difference between CHX and IONPs-CS-CHX156 regarding lactic acid quantification, but these compounds resulted in substantial reductions of 94.52 (*p* < 0.001) and 93.86% (*p* < 0.001) compared to NC, respectively (Figure 1D).

### 2.2. Oral Candidiasis Microcosm Biofilm

**Figure 2:**
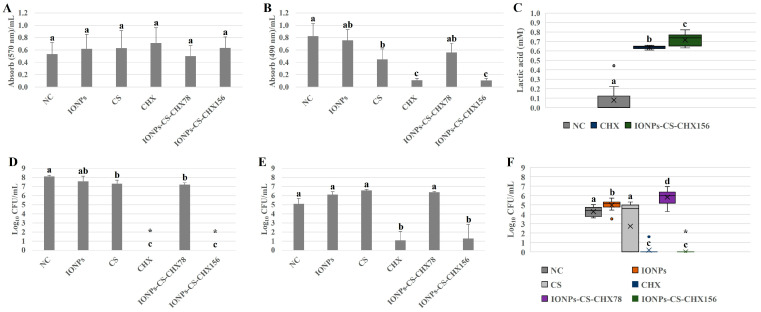
Semi-quantitative and quantitative assays of total biomass (**A**), metabolism (**B**), lactic acid production (**C**), and loads of bacteria (**D**), *Candida glabrata* (**E**), and *Candida albicans* (**F**) of oral candidiasis microcosm biofilms in the various experimental groups. Seventy-two-hour biofilms remained in contact with the following agents for 24 h: pure McBain medium (negative control (NC)), iron oxide nanoparticles (IONPs) and chitosan (CS), both at 218.75 µg/mL, chlorhexidine at 312 μg/mL (CHX), and nanocarrier loaded with CHX at 78 (IONPs-CS-CHX78) or 156 μg/mL (IONPs-CS-CHX156). * Indicates complete eradication of cultivable cells. ° Represents the outliers in images C and F. Groups differ by distinct lowercase letters (one-way ANOVA or Kruskal–Wallis test and Student-Newman-Keuls post hoc method; *p* < 0.05).

Total biomass was not significantly affected after exposure of oral candidiasis microcosm biofilms to the different agents (Figure 2A). For metabolic activity, however, a different trend was noted (Figure 2B). While CS induced a 45.78% reduction in metabolism compared to NC (*p* = 0.031), similar and expressive reductions of 86.74% (*p* < 0.001) were found after the application of CHX and IONPs-CS-CHX156 (Figure 2B). In contrast, CHX and IONPs-CS-CHX156 led to significant increases in lactic acid concentration of 9- and 10.14-fold, respectively, compared to NC (*p* < 0.001; Figure 2C). Furthermore, CHX and IONPs-CS-CHX156 were statistically different, with a higher lactic acid concentration for the biofilm treated with the nanocarrier (Figure 2C).

For total bacterial load, CHX and IONPs-CS-CHX156 exhibited substantial 8.13-log_10_ (*p* < 0.001) decreases compared to NC, with no significant difference between the two agents (Figure 2D). Additionally, when the biofilm was exposed to CS and IONPs-CS-CHX78, reductions of 0.82- (*p* = 0.035) and 0.92-log_10_ (*p* = 0.032) relative to NC were verified, respectively (Figure 2D). As for *C. glabrata* quantification from the microcosm biofilm, only CHX and IONPs-CS-CHX156 demonstrated a reducing effect relative to NC, generating significant decreases of 4- (*p* < 0.001) and 3.81-log_10_ (*p* < 0.001), respectively (Figure 2E). For *C. albicans*, while IONPs and IONPs-CS-CHX78 increased (0.7- to 1.55-log_10_; *p* < 0.05) the fungal burden compared to NC, CHX and IONPs-CS-CHX156 induced significant reductions (4.09- to 4.27-log_10_; *p* < 0.001; Figure 2F).

**Figure 3:**
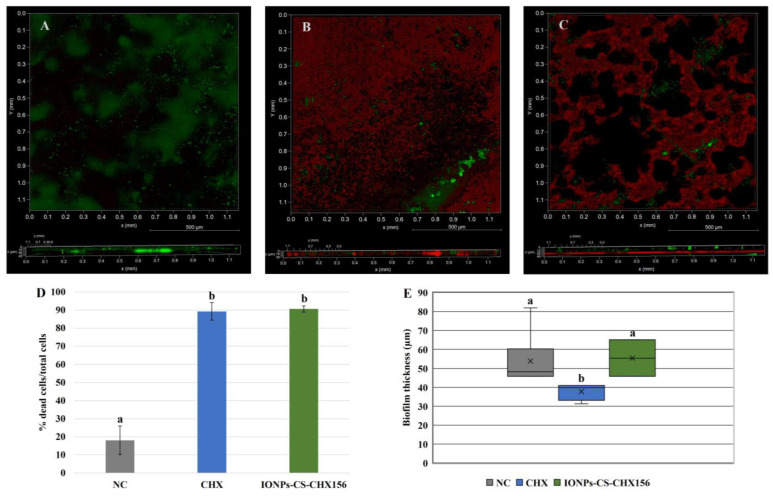
Qualitative (**A**–**C**), cell viability (**D**), and thickness (**E**) analyses of oral candidiasis microcosm biofilms by confocal microscopy. Seventy-two-hour biofilms were exposed to the following agents for 24 h: pure McBain medium (negative control (NC); image A), chlorhexidine at 312 μg/mL (CHX; image B), and chitosan (CS)-coated iron oxide nanoparticles (IONPs) carrying CHX at 156 μg/mL (IONPs-CS-CHX156; image C). Differences among groups are shown by distinct lowercase letters (one-way ANOVA or Kruskal–Wallis test and Student-Newman-Keuls post hoc method; *p* < 0.05). Green and red areas in images A, B, and C show live and dead microbial cells, respectively.

Confocal microscopy-based analysis of biofilm architecture revealed clusters of microbial cells partially covering the surfaces, especially for the NC (Figure 3A) and IONPs-CS-CHX156 (Figure 3C) groups. In the NC (Figure 3A), a predominance of live cells (green fluorescence) was observed, whereas red fluorescence (dead cells) predominated in the CHX and IONPs-CS-CHX156 groups, although there were small foci of live cells (Figure 3B,C). The mean percentages of dead cells for biofilms treated with CHX and IONPs-CS-CHX156 were 89.26 and 90.63%, respectively, and these values were statistically distinct from those found for the NC (18.11%; *p* < 0.001; Figure 3D). Finally, biofilm treatment with CHX resulted in the lowest biofilm thickness value, revealing a significant difference relative to the other groups (*p* = 0.003; Figure 3E).

## 3. Discussion

Antimicrobial resistance is a serious public health threat, as microorganisms evolve and develop the ability to withstand treatments that were once effective against them [29]. Recurrent manifestations of oral candidiasis are linked to microbial resistance, hosts with compromised immune responses, and the use of dentures, the latter being the main cause of creating a conducive environment to *Candida* spp. multiplication and biofilm formation. Furthermore, inefficient oral hygiene contributes to the recalcitrance of fungal infection [30]. Within this context, the current study assessed the antibiofilm effects of a CHX carrier system based on CS-coated IONPs as a therapeutic alternative for the management of oral candidiasis. Overall, both hypotheses of this study were partially accepted. The nanocarrier reduced some microcosm biofilm parameters, particularly when loaded with CHX at 156 µg/mL. Moreover, the nanocarrier (IONPs-CS-CHX156) and free CHX exhibited similar antibiofilm effects in most of the parameters tested.

For the microcosm biofilm without *Candida* spp., which served as the control biofilm model, treatments with nanocarriers showed dose-dependent efficacy in reducing cellular metabolism and total bacterial load (Figure 1). Furthermore, IONPs-CS-CHX156 led to an expressive reduction in lactic acid levels (Figure 1). Thus, it can be inferred that the reductions in metabolism and lactic acid induced by the nanocarrier are related to the cell death observed in the bacterial load enumeration assay. Interestingly, although IONPs-CS-CHX156 and CHX eliminated microbial cells in the colony-forming unit (CFU) assay, these agents did not produce 100% reductions in cellular metabolism and lactic acid production. These findings suggest that some viable microbial cells may not have been cultivable on the agar medium used for CFU quantification. Evidence in the literature indicates that the antimicrobial action of IONPs is related to their small size and large surface area, which facilitates adhesion to bacterial cells. This interaction triggers the release of metal ions, inducing oxidative stress through free radical formation. The integrity of the microbial cell membrane is also damaged, generating ruptures that lead to cell death [31]. Despite the biocidal potential of IONPs, the current study demonstrated that nanoparticles administered alone did not significantly reduce the analyzed variables, which may be attributed to two factors. First, the concentration used (218.75 µg/mL), based on the nanocarrier formulation, was lower than the concentration reported in the literature as necessary to achieve an antifungal effect against *C. albicans* (500 µg/mL) [32]. Second, the IONPs administered alone lacked functionalization, since a previous study has shown that CS coating enhances the effect of IONPs on bacterial biofilms [33]. On the other hand, both CS and CHX significantly reduced the metabolism and microbial load in the microcosm biofilm (Figure 1). The amine group of CS, which is positively charged, interacts with the surfaces of bacterial cells, which acquire a negative charge when in suspension. This process alters cell permeability and osmotic balance, causing the release of intracellular ions and proteins into the extracellular environment [34]. CHX operates through a comparable mechanism, as its cationic charge damages microbial cell structures, leading to cytoplasmic coagulation and precipitation, depending on exposure time and compound concentration [35,36].

Considering that IONPs-CS-CHX156 was comparable to CHX but more effective than IONPs-CS-CHX78 and the untreated controls, it is evident that the effect of the nanocarrier on the salivary microcosm biofilm without *Candida* spp. depends on the dose of CHX loaded. This effect reflects the combined mechanisms of action of CS and CHX, without direct involvement of IONPs in microbial elimination. However, as the nanocarrier containing 156 µg/mL CHX exhibited the same efficacy as CHX at twice the concentration (312 µg/mL), it is reasonable to infer that IONPs acted exclusively as carriers, facilitating CHX penetration and antimicrobial action throughout the biofilm layers, even at a lower concentration. These findings indicate that the main advantage of the nanocarrier lies in its CHX dose-sparing effect rather than in enhanced antibiofilm efficacy. Consequently, this nanotherapy may reduce adverse effects associated with high CHX concentrations while maintaining antibiofilm activity comparable to that of the free antiseptic.

For total biomass, however, none of the experimental groups showed significant reductions compared to NC (Figure 1A). These data may be associated with the characteristics of the crystal violet (CV) reagent, a positively charged basic dye that binds to negatively charged components, including cells and the extracellular matrix of biofilms. Furthermore, CV does not distinguish between live and dead cells within the biofilm community. The dye may also bind nonspecifically to negatively charged molecules, leading to inconsistent dye extraction from biofilms when the decolorizing solution is applied [37]. This could explain the biomass results found, despite the reductions detected in metabolism, microbial load, and lactic acid production.

When *C. albicans* and *C. glabrata* were introduced into the polymicrobial biofilm to simulate an oral candidiasis microcosm, similar outcomes to those of the control biofilm model were observed for all variables investigated, except for lactic acid production (Figure 2). Thus, IONPs-CS-CHX156 and 312 µg/mL CHX were comparable to each other and were the compounds with the greatest antibiofilm potential, even increasing the diversity and variety of microorganisms present in the community. The explanations for the nanocarrier effects on the biomass, metabolism, and bacterial load of the oral candidiasis microcosm biofilm follow the same line of reasoning applied to the control biofilm model, with a small particularity for the fungal load. The *Candida* spp. CFU counts (Figure 2E,F) were not reduced after contact with IONPs and CS applied in free form. Thus, the antifungal action of the IONPs-CS-CHX156 carrier system on *C. albicans* and *C. glabrata* depends on the presence of CHX, with the participation of IONPs-CS facilitating the transport and penetration of the antiseptic. Although this study did not evaluate the CHX release kinetics from the nanocarrier, a previous study reported a release rate of 12.09% from the IONPs-CHX system after 4 h [25]. Considering that biofilms in the present study were exposed to the nanocarrier for 24 h, it is reasonable to infer that CHX release was greater and progressive, which may have favoured antibiofilm action even at half the concentration of the control, due to the prolonged availability of the antimicrobial. It is also noteworthy that the highest CHX concentration evaluated in the nanocarrier (156 µg/mL) is lower than that frequently found in commercial mouthwashes (0.12% or 1200 µg/mL). Nevertheless, the 156 µg/mL concentration may be relevant for clinical applications, especially for a nanocarrier capable of sustaining CHX release while maintaining antibiofilm efficacy.

Interestingly, treatments of oral candidiasis biofilm with IONPs-CS-CHX156 and CHX significantly reduced the *C. glabrata* burden (Figure 2E) and completely eradicated the number of cultivable *C. albicans* cells (Figure 2F). From a clinical perspective, these data are encouraging because *C. albicans* is the microorganism most regularly isolated from oral candidiasis patients. Due to its antibiofilm effect being conditioned by the presence of CHX, it is likely that the nanocarrier increased membrane permeability and triggered modifications in the cell wall of *C. albicans* [15]. This may have increased the intracellular concentration of metal ions from the extracellular environment, stimulating the production of ROS, which are toxic to fungal cells. The oxidative stress generated may have also been responsible for stimulating apoptosis of *C. albicans* cells [15].

The evidence of viability of the oral candidiasis microcosm biofilm by confocal microscopy corroborates the CFU and metabolic activity data, since the IONPs-CS-CHX156 and CHX groups significantly reduced cell viability (Figure 3D). However, the analysis of biofilm thickness showed a different outcome, indicating that the second hypothesis was not fully accepted. While CHX led to a significant reduction, the IONPs-CS-CHX156 nanocarrier did not differ from the NC (Figure 3E). These findings suggest that free CHX interacts more readily with the extracellular matrix than the nanocarrier, which disrupts the microcosm biofilm. The conjugation of CHX with IONPs-CS may have created a compound with a larger volume, hindering the direct interaction of the carried compound with the main components of the extracellular matrix (polysaccharides and proteins). Furthermore, the amount of CHX released from the nanosystem may have been sufficient to kill the cells but insufficient to disrupt the physical structure of the biofilm.

The increased *C. albicans* burden after exposure to IONPs alone and IONPs-CS-CHX78 (Figure 2F) is also a relevant finding that could be attributed to the hormesis effect [38]. Thus, the nanocarrier at its lowest dose and IONPs at subinhibitory concentrations boosted fungal growth and survival. These compounds may have generated chemical or molecular modifications in the biofilm, potentially affecting mechanisms like quorum sensing. Such changes in community balance likely facilitated the development of *C. albicans*. Therefore, IONPs or subinhibitory concentrations of CHX in the carrier could exacerbate the clinical presentation of oral candidiasis, demonstrating that antibiofilm efficacy is directly dependent on the administered dose.

Regarding the lactic acid quantification, the oral candidiasis microcosm biofilm showed reduced acidity compared to the control model biofilm (Figure 1D and Figure 2C). This result may be attributed to the presence of fungal species in the candidiasis model, which may have consumed the lactic acid produced by acidogenic bacteria as an energy source for their maintenance, resulting in very low levels of lactic acid. This hypothesis is supported by previous literature demonstrating that *C. albicans* in mixed biofilms with *Streptococcus mutans* contributes to making the biofilm less acidic over time, probably decreasing the capacity of the dual biofilm to form carious lesions [39,40]. For the oral candidiasis microcosm biofilm, treatments with the nanocarrier and CHX alone resulted in significantly higher lactic acid levels than those found for the NC (Figure 2C). A microbial shift in community composition is likely responsible for the increased lactic acid levels. The elimination of many bacteria and *C. albicans* may have freed up nutrients and space for the remaining *C. glabrata* cells and acidogenic bacteria, reducing interspecies competition. Consequently, these surviving cells may have altered their behaviour, increasing their metabolism and producing greater amounts of lactic acid as a survival mechanism and adaptation to the new biofilm ecosystem. Specifically for the microcosm biofilm model of oral candidiasis tested, these findings suggest that biofilm pathogenicity increases after exposure to CHX and IONPs-CS-CHX156, despite reductions in cell load and overall metabolism. Clinically, elevated lactic acid levels can facilitate the growth of acidogenic and aciduric microorganisms, contributing to the development of carious lesions. Furthermore, high acid levels may exacerbate denture stomatitis and cause damage to the denture base resin. Future research should more deeply analyze the microbial composition and metabolism of the remaining biofilm cells after treatment with the nanocarrier, including the identification of possible genes associated with acid production, especially in *C. glabrata*.

When the same nanocarrier used in the present study was tested on simpler biofilm models (mono- or dual-species), it was significantly effective in reducing biomass, metabolism, and number of cultivable cells [27,28]. Furthermore, when administered at low concentrations, the nanocarrier mitigated CHX toxicity on murine fibroblasts [28]. Comparing the findings of the current investigation with previous studies reveals that the IONPs-CS-CHX nanocarrier remains effective even against more complex polymicrobial biofilm models, such as the oral candidiasis microcosm formed in an active adhesion model. This model is more robust and representative of real-world oral conditions, as it better reproduces the interactions between microbial species. A key novel finding, however, was the unexpected increase in lactic acid production within these polymicrobial biofilms after nanocarrier exposure.

In light of these results, this nanocarrier could be clinically applied as a solution for eliminating or inhibiting pathogenic microorganisms in oral tissues (antisepsis) or on prosthetic device surfaces (disinfection). It is noteworthy that the 24 h exposure period used in this study was much longer than the clinically typical duration of a few minutes for CHX mouthwashes. This continuous exposure, however, allowed for a direct comparison with previously published studies that used similar protocols [27,28]. Additionally, the nanocarrier could be used as a disinfectant solution for immersing removable dentures during nocturnal sleep or as a coating for dental appliances, demonstrating the importance of prolonged exposure periods.

Considering that the properties of the nanocarrier were obtained from an in vitro biofilm model with limited donor variability, before this new therapy can be applied clinically, further investigations are necessary. Future studies should investigate (i) the impacts of the nanocarrier on microcosm biofilms formed with clinical isolates of *Candida* after shorter treatment periods, (ii) the detailed microbial composition of the microcosm biofilm by genetic sequencing after contact with the nanocarrier, (iii) its in vivo effectiveness in oral candidiasis models induced in rats, as well as (iv) its actual clinical effectiveness and safety (toxicity and tissue penetration). All these investigations are necessary to overcome the limitations of the present study and to make progress in deepening the knowledge of the nanocarrier potential in the treatment of oral candidiasis.

## 4. Materials and Methods

### 4.1. Assembly and Analysis of the IONPs-CS-CHX Nanocarrier

For the fabrication of the IONPs-CS-CHX carrier system, a colloidal suspension of IONPs (*nChemi*, São Carlos, São Paulo, Brazil) and a CS (Sigma-Aldrich; CAS n° 9012-76-4; medium molecular weight) suspension were prepared at a concentration of 1400 µg/mL. An equal volume of each suspension was mixed, as detailed in a previous study [27]. Subsequently, the CHX powder (Sigma-Aldrich; CAS n° 55-56-1) was incorporated into the IONPs-CS system and stirred for 1 h to reach a concentration of 500 µg/mL [27]. The IONPs-CS-CHX nanocarrier was analyzed by transmission electron microscopy, X-ray diffraction, Fourier transform infrared spectroscopy, and thermogravimetric analysis. Physicochemical tests showed that the nanocarrier has a spherical configuration and a diameter of 33.6 ± 10.7 nm [27]. Furthermore, the crystal structure of IONPs remained unaltered after the incorporation of CS and CHX [27]. To confirm the conjugation, the presence of the main CHX functional groups was identified in the nanocarrier. Thermal analysis, in turn, showed a mass loss difference of approximately 9% between the IONPs-CS and IONPs-CS-CHX compounds, corresponding to the mass of CHX conjugated to the carrier system [27]. Together, these analyses validated the conjugation of CHX and the formation of the IONP-CS-CHX nanocarrier. 

### 4.2. Human Saliva Collection

Before obtaining human saliva, the current study obtained approval from the Human Research Ethics Committee of the Araçatuba School of Dentistry (FOA/UNESP), process CAAE 81680024.1.0000.5420. Three adults (two men and one woman) consented to participate in the study and received prior instructions related to the saliva collection step, as specified in another study [41]. The randomly selected donors, who were free of systemic or oral diseases, were aged 27, 28, and 43. The selection of three donors aimed to represent the microbial diversity of the population and minimize the impact of the salivary composition of a single individual on biofilm formation. On the day of sample collection, each volunteer was instructed to chew a strip of Parafilm^®^ (Sigma-Aldrich, St. Louis, MO, USA) to stimulate saliva production. As saliva accumulated in the mouth, donors expelled it into 50 mL centrifuge tubes, which were kept in contact with ice. Subsequently, the saliva samples were diluted (1:1) in 60% glycerol, aliquoted into polypropylene microtubes, and frozen at −80 °C.

### 4.3. Fungal Strains

In one of the models of polymicrobial biofilms, two *Candida* species from the American Type Culture Collection (ATCC) were used to simulate an oral candidiasis microcosm: *C. albicans* ATCC 10231 and *C. glabrata* ATCC 90030. Suspensions of these fungal strains preserved at −80 °C were thawed and reactivated on Sabouraud Dextrose Agar (Difco, Difco Laboratories, Becton, Dickinson and Company, Le Pont-de-Claix, France). After 24 h of incubation in aerobic conditions at 37 °C, colonies of each fungal species were separately placed in conical flasks with 30 mL of Sabouraud Dextrose Broth (Difco). These flasks were kept overnight on a shaker (120 rpm) with controlled temperature (37 °C), under aerobic conditions. To obtain the final inoculum of each strain, the previously prepared fungal suspensions were processed and adjusted to 1 × 10^7^ cells/mL in McBain culture medium [42] containing 0.2% sucrose, following a previously published protocol [41].

### 4.4. Development of Microcosm Biofilms and Exposure to the Nanocarrier

The microcosm biofilms were developed on the surfaces of 13 mm diameter glass discs (OLEN; Kasvi, Pinhais, Paraná, Brazil), which were attached to the steel lid of the Amsterdam Active Attachment (AAA) model [43]. This model allowed the vertical positioning of the glass discs within the wells of 24-well cell culture plates.

Two different polymicrobial biofilm models were developed, one without *Candida* species and the other with fungal species. For the salivary microcosm biofilm without *Candida* species, which served as a control model, a microbial suspension was prepared by mixing one part of the combined saliva from the three donors with 50 parts of McBain culture medium containing 0.2% sucrose. This suspension (1.5 mL) was then inserted into the wells of a 24-well plate (Falcon^®^; Corning Incorporated-Life Sciences, New York, NY, USA), and the AAA model steel lid was used to close this plate. The plate + AAA model set remained in a microbiological incubator for 8 h at 37 °C (5% CO_2_). Then, a new 24-well plate was filled with 1.5 mL of McBain medium (pure and fresh), and the lid of the AAA model, which contained the microbial cells adhered to the glass discs, was used to close this plate. After a new incubation period (16 h; 5% CO_2_) at 37 °C, the lid of the AAA model was relocated to a fresh 24-well plate filled with McBain medium containing 0.2% sucrose. This last set (plate + lid of the AAA model) was kept in an incubator (5% CO_2_, 37 °C) for another 48 h. Following the initial 24 h, the culture medium was refreshed. Consequently, 72 h salivary microcosm biofilms were developed.

For the growth of salivary microcosm biofilm with *Candida* species, aiming to simulate an oral candidiasis microcosm, the procedures were performed as described above for the control model. The only variation was that, after the initial 24 h of biofilm development, the lid of the AAA model was relocated to a fresh 24-well plate filled with fungal inoculum (1 × 10^7^ cells/mL of *C. albicans* + 1 × 10^7^ cells/mL of *C. glabrata* in McBain medium). This new plate remained in the incubator (5% CO_2_; 37 °C) for another 48 h, thus completing 72 h of biofilm growth.

In the exposure phase of the biofilms to the nanocarrier, the colloidal suspension of the IONPs-CS-CHX compound (IONPs-CS at 700 µg/mL + CHX at 500 µg/mL) was diluted in McBain medium containing 0.2% sucrose, resulting in two concentrations of CHX in the nanocarrier: 78 (IONPs-CS-CHX78) or 156 µg/mL (IONPs-CS-CHX156). These doses are 50- and 100-fold higher than the lowest minimum inhibitory concentration (1.56 µg/mL) recorded for the fungal strains examined in the present study [27,28]. A volume of 1.5 mL of each nanocarrier suspension was introduced into the wells of a fresh 24-well plate. The polystyrene plate was then sealed with the AAA model lid, which contained the 72 h biofilms developed on the glass discs. The set remained for 24 h in a microbiological incubator at 37 °C (5% CO_2_). In addition to the nanocarrier, the individual components were evaluated in three separate groups: 218.75 µg/mL IONPs, 218.75 µg/mL CS, and 312 µg/mL CHX. The concentrations of free IONPs and CS were chosen to match their respective doses in the highest concentration nanocarrier formulation (IONPs-CS-CHX156). Free CHX at 312 µg/mL served as a high-dose control to determine if the nanocarrier, with half the CHX concentration (156 µg/mL), could achieve a comparable or superior antibiofilm effect. The biofilms were also exposed to pure McBain medium with 0.2% sucrose, constituting the NC.

### 4.5. Semi-Quantitative and Quantitative Analysis of Biofilms

After exposing the biofilms to various agents, the glass discs were washed with phosphate-buffered saline (PBS; 0.1 M, pH 7.0), and assays were conducted to verify the antibiofilm effects. To evaluate total biomass production, biofilms were fixed for 15 min with 99% methanol (Sigma-Aldrich), dried at room temperature, and stained for 5 min with 1% CV (Sigma-Aldrich) [44]. The biofilms were then placed in contact with a 33% acetic acid solution (Sigma-Aldrich) to release the dye [44]. The absorbance of the resulting solutions for each experimental group was read at 570 nm. To obtain the final result, the absorbance values of the experimental groups were subtracted from the blank values (glass discs without biofilm exposed to McBain culture medium).

The XTT (2,3-(2-methoxy-4-nitro-5-sulphophenyl)-5-[(phenylamino)carbonyl]-2H-tetrazolium hydroxide; Sigma-Aldrich) reduction assay was used to determine biofilm cell metabolism, following the protocol of a previous study [44]. Briefly, biofilms were left in contact with a solution of XTT (150 mg/L) + phenazine metasulfate (10 mg/L) for 3 h (37 °C; 120 rpm) in the dark [44]. The absorbance of the solutions was then read at 490 nm. Blanks were also included, as detailed for the biomass assay.

CFU counts were used to quantify bacterial and *Candida* loads, as previously detailed [41]. The biofilm-containing glass discs were placed in tubes containing PBS and vortexed. After biofilm detachment, the microbial suspensions were diluted in PBS and plated on Trypticase Soy Agar (TSA; Difco) enriched with sheep blood, glucose, hemin, menadione, and 7 µg/mL amphotericin B for bacterial quantification. For *C. albicans* and *C. glabrata* counts, the suspensions were grown on CHROMagar *Candida* (Difco) [41]. TSA plates were incubated at 5% CO_2_, while the CHROMagar *Candida* plates were incubated aerobically [41]. CFUs were counted after 24–48 h of incubation at 37 °C.

Regarding lactic acid production, the NC and the experimental groups that showed the best performance in other quantitative tests (CHX and IONPs-CS-CHX156) were selected for analysis. The remaining groups were excluded due to their minimal antibiofilm effects. Briefly, the biofilms were placed in buffered peptone water (BPW) enriched with 0.2% glucose and incubated for 3 h at 5% CO_2_. Next, the enzyme Lactate Dehydrogenase (Sigma-Aldrich) was pipetted into the samples, and the absorbance was subsequently recorded at 340 nm. The lactate concentrations in the samples were then determined using a standard curve of sodium L-lactate (Sigma-Aldrich) [45].

For each parameter analyzed, three independent assays were conducted, each in triplicate (n = 9).

### 4.6. Biofilm Viability and Thickness by Confocal Laser Scanning Microscopy

This analysis was conducted for the microcosm biofilm containing *C. albicans* and *C. glabrata*. Biofilms were formed and exposed to the treatment groups (i) IONPs-CS-CHX156, (ii) CHX at 312 µg/mL, and (iii) McBain medium (NC), as detailed above. The LIVE/DEAD^®^ BacLight™ kit (Invitrogen, Carlsbad, CA, USA) was used to stain the biofilms. The number of dead cells in each experimental group was estimated based on the red and green fluorescence intensities, as reported in another study [41]. To determine the thickness of each biofilm, three-dimensional images (3D-thickness) were produced, and the values provided by the equipment software were recorded. In total, two biofilm samples from each group and three distinct areas of each sample were evaluated (n = 6) in the STELLARIS 5 confocal microscope (Leica, Wetzlar, Germany).

### 4.7. Statistical Analysis

The data were subjected to the Shapiro–Wilk and Levene’s tests, respectively, for normality and homoscedasticity analyses. In general, these assumptions were met, except for the data on lactic acid (for both types of polymicrobial biofilm), fungal load of *C. albicans*, and biofilm thickness. Both parametric and nonparametric data sets were examined by one-way analysis of variance (ANOVA) and Kruskal–Wallis test, respectively. For all statistical analyses, the Student–Newman–Keuls post hoc method was applied, using a two-tailed approach. Data analysis was conducted with SigmaPlot software (version 12.0; Systat Software Inc., San Jose, CA, USA), adopting a significance level of 5%.

## 5. Conclusions

Based on the results of this investigation, the CHX nanocarrier has a significant effect on reducing the metabolic activity, microbial load, and cell viability of oral candidiasis microcosm biofilms, especially when carrying CHX at 156 µg/mL. Notably, the nanocarrier showed the same antibiofilm effect as non-carried CHX, but at half the concentration. This suggests that the key benefit of the nanotherapy lies in reducing the required CHX dose, thereby potentially mitigating side effects, rather than providing superior antibiofilm action. However, the nanocarrier also increased lactic acid production within the oral candidiasis microcosm biofilm, which may enhance the pathogenicity of the remaining cells.

## Data Availability

The original contributions presented in this study are included in the article. Further inquiries can be directed to the corresponding author.

## Abbreviations

The following abbreviations are used in this manuscript:

IONPs	Iron oxide nanoparticles
CS	Chitosan
CHX	Chlorhexidine
NC	Negative control
ROS	Reactive oxygen species
ATCC	American Type Culture Collection
AAA	Amsterdam Active Attachment
CV	Crystal violet
CFU	Colony-forming unit
PBS	Phosphate-buffered saline
XTT	(2,3-(2-methoxy-4-nitro-5-sulphophenyl)-5-[(phenylamino)carbonyl]-2H-tetrazlium hydroxide)
TSA	Trypticase soy agar
BPW	Buffered peptone water
